# The ideology of religious stigmatization: imposed identities as a tool for constructing the national community in Kazakhstan

**DOI:** 10.3389/fsoc.2026.1805651

**Published:** 2026-09-09

**Authors:** Tussubzhan Duzbayev, Essenzhol Aliyarov, Natalya Seitakhmetova, Sholpan Zhandossova, Marhabbat Nurov

**Affiliations:** 1 Department of Political Science and Political Technologies, Al-Farabi Kazakh National University, Almaty, Kazakhstan; 2 Kazakhstan Center for Humanitarian and Political Trends, Almaty, Kazakhstan; 3 Institute of Philosophy, Political Science and Religious Studies, CS MSHE RK, Almaty, Kazakhstan; 4 Higher School of International Relations and Diplomacy, Turan University, Almaty, Kazakhstan

**Keywords:** hegemony, imposed identities, Kazakhstan, nation-building, religious minorities, religious stigma, securitization of religion, state ideology

## Abstract

**Introduction:**

This study examines how imposed religious identities operate as an ideological instrument in Kazakhstan’s post-Soviet transformation, with particular attention to the social production of stigma and the ways in which boundaries of the national community are articulated in public discourse.

**Methods:**

Building on Erving Goffman’s concept of stigma and critical approaches to ideology and governance (Althusser, Gramsci, and Foucault), the article adopts an interdisciplinary case-study design combining descriptive analysis of secondary quantitative data with qualitative analysis of key legal and policy documents and a purposively selected set of six publicly accessible media and official materials published between 2016 and 2023.

**Results:**

The analysis identifies changes in patterns of religious self-identification alongside differentiated discursive positioning of religious groups. Within the reviewed materials, “traditional” confessions, particularly Hanafi Islam and Orthodox Christianity, are associated with cultural heritage and social stability, whereas some movements categorized as “non-traditional” are linked to security-related and “foreign influence” narratives. These qualitative patterns are especially relevant to new religious movements, denominations perceived as non-traditional in the region, and youth whose religious learning is mediated by digital and transnational networks.

**Discussion:**

The study concludes that formal commitments to freedom of religion and pluralism coexist with practices of differentiated regulation and representation that can contribute to the stabilization of confessional hierarchies within broader processes of nation-building.

## 1 Introduction

Contemporary societies are shaped by complex processes of social categorization and identification, within which certain groups are ascribed negative characteristics that generate what has been described as a form of “social curse” (Kellezi et al., 2019). Goffman (1963) traced the term “stigma” to bodily signs used in ancient Greece to mark individuals regarded as morally discredited – such as slaves, criminals, or traitors. He subsequently developed the concept to describe attributes that discredit individuals within particular social relationships and reduce them, in the eyes of others, from a whole person to a socially devalued one. Over time, the concept of stigma has evolved, shifting the emphasis from physical signs to the experience of shame itself and to changing modalities of social exclusion.

Within Kazakhstan’s multiethnic and multiconfessional setting, studies by Kazakhstan-based scholars of ethno-religious identity and religious risk, together with analyses of interfaith harmony and cultural transformation, provide an important contextual backdrop for examining how religious categories become socially and politically consequential (Muhambetaliev et al., 2015; Kamalova et al., 2024).

Within the religious domain, the phenomenon of imposed identities carries particular ideological significance, since religious affiliation remains one of the key markers of social identity, shaping access to resources, social status, and opportunities for participation in public life (Fox, 2018). Moreover, religious identities are mobilized by the state as an instrument for delineating the boundaries of the national community and legitimizing a particular ideological model of development.

The post-Soviet space, including Kazakhstan, constitutes a distinctive laboratory for examining the construction and imposition of religious identities amid radical transformations of ideological and confessional landscapes. Following the dissolution of the Soviet Union and the erosion of the dominant atheist ideology, Kazakhstan faced the task of articulating a new national ideology in which religion has assumed an ambivalent role (Yemelianova, 2014; Dave, 2007).

From a macro-sociological perspective, these developments can be situated within broader accounts of systemic transformation and modernization pressures in complex societies (Parsons, 1971; Parsons, 1998), alongside Kazakhstan-based scholarship examining the post-Soviet reconfiguration of state–religion relations, religious institutions, and identity (Beisenov and Igisenova, 2018; Burova et al., 2020).

This process has entailed both the revival of “traditional” religious practices and the emergence of new denominations and movements, resulting in a complex and multi-layered religious landscape (Laruelle, 2018; Yemelianova, 2014). Yet alongside the expansion of religious pluralism, one can observe an intensification of ideological stigmatization directed at religious groups, which are constructed as incompatible with national ideology or as threatening its foundations.

The relevance of this problematic has grown further in the context of global processes of the securitization of religion, whereby religious belonging is increasingly interpreted through the lens of security and ideological loyalty, and certain denominations and currents are routinely associated with terrorism and extremism (Omelicheva, 2011). In Kazakhstan, state ideology thus seeks to balance the constitutional principle of freedom of religion with the perceived necessity of regulating the religious sphere to preserve ideological cohesion and ensure national security.

Against this backdrop, the present study aims to identify the ideological mechanisms through which religious identities are constructed and imposed in contemporary Kazakhstan, to analyze their role in the formation of national ideology, and to consider their potential implications for groups subjected to stigmatizing classifications. The study addresses the following questions:

Which religious groups are positioned in public and institutional discourse as targets of negative identity construction, and what ideological functions do these classifications perform?

How do processes of religious stigmatization contribute to the formation and consolidation of national ideology?

What roles do the media, state policy, and public discourse play in the ideological construction of religious stigma?

How are transnational religious identities framed and perceived as an ideological threat to the nation-state?

What ideological contradictions emerge from attempts to combine religious revival with a secular state and modernization?

More broadly, the study treats religion as a social and cultural phenomenon shaped by collective meanings and institutional arrangements: as a “social fact” embedded in moral communities (Durkheim, 2001), as a source of culturally mediated symbols and interpretive frameworks (Geertz, 1973), and as a domain in which religious ideas may intersect with wider patterns of authority and social organization (Weber, 2002). In addition, the analysis is attentive to the socially constructed character of taken-for-granted realities and public classifications, including those applied to religious groups (Berger and Luckmann, 1995). Where Russian-language editions are referenced for terminological consistency, established translations are used (Geertz, 2004).

The theoretical framework draws on classical sociological accounts of stigma (Goffman, 1963), critical theories of ideology (Althusser, 1971; Gramsci, 1971; Foucault, 1991), contemporary conceptualizations of “social curse” (Kellezi et al., 2019), theories of the securitization of religion (Omelicheva, 2011), and post-secular approaches to religion in the public sphere (Habermas, 2006; Taylor, 2007).

This security-oriented framing has also been discussed in relation to narratives of “radical Islam” in the wider Central Asian context, where political and media discourse may conflate diverse currents under generalized threat labels (Naumkin, 2005).

At the same time, a substantial body of ethnographic scholarship on lived religious practice in the wider Central Asian region cautions against treating official “traditional/non-traditional” classifications as straightforward descriptions of how believers themselves experience and negotiate their faith. Studies of Islam in neighboring Uzbekistan and Kyrgyzstan show that ordinary believers construct their religious identities through everyday moral reasoning, family history, and local custom rather than through the binary categories privileged in security and media discourse (Rasanayagam, 2011; McBrien, 2017). For Kazakhstan specifically, earlier historical-ethnographic work on the persistence of vernacular Islamic practice and collective memory through the Soviet period offers an important, and currently underused, baseline for evaluating how far contemporary official categories of “traditional” religiosity correspond to, or depart from, longer-standing local religious practice (Privratsky, 2001). Relatedly, recent ethnographic work on the broader Islamic revival movements of Central Asia examines how Soviet-era secular categories continue to shape what counts as legitimate religious practice, and how a non-observant, “everyday” form of Islam persists alongside more visibly “traditional” or “revivalist” currents (Boron, 2024). This literature is directly relevant to the transnational concerns discussed in §1.7: it suggests that the “non-traditional” label applied to some Islamic currents in Kazakhstan often marks not doctrinal deviance as such, but visible departure from a Soviet-shaped norm of low-observance religiosity, which is precisely the kind of departure that transnational networks and renewed religious practice make more visible. Engaging with this literature does not close the empirical gap addressed in the Discussion (§4.3), but it situates the present study’s account of “traditional” versus “non-traditional” Islam within a wider anthropological conversation about the constructed nature of such categories in the post-Soviet space.

### 1.1 Theoretical foundations for the study of imposed religious identities

#### 1.1.1 The concept of stigma and “social curse”

A foundational framework for understanding imposed identities is Erving Goffman’s concept of stigma (Goffman, 1963). Goffman defined stigma as an attribute that discredits an individual in the eyes of society, transforming them from a “normal” person into one who is “tainted” or “discredited.” In the religious sphere, stigma may be associated with affiliation with a particular denomination, the performance of specific rituals, or adherence to religious beliefs that diverge from those dominant in each society.

Goffman distinguished three types of stigma: physical deformities, blemishes of individual character, and “tribal stigma” linked to race, nationality, and religion (Goffman, 1963). Religious stigma belongs to this latter category and is distinctive in that it can be transmitted across generations and extend to all members of the stigmatized group regardless of their individual traits or conduct.

Building on social identity research, Kellezi et al. (2019) show that shared group identities can function both as sources of support and meaning (“social cures”) and as sources of burden, ostracism, and distress (“social curses”). In the present article, this insight is used cautiously to consider how group-based religious categorization may compound the effects of stigma; it is not treated as a separate theory of collective religious stigmatization.

### 1.2 The securitization of religion and the construction of threat

In the post-Soviet period – particularly in the aftermath of the events of 11 September 2001 – religion has increasingly been interpreted through the lens of security (Omelicheva, 2011). The securitization of religion entails a reframing of religious phenomena as potential threats to national security, thereby legitimizing extraordinary measures of surveillance, control, and the curtailment of religious freedoms.

Across Central Asia, including Kazakhstan, the growing public and political role of Islam has frequently been interpreted through security-oriented debates concerning Islamism, extremism, and political stability (Laruelle, 2013; Omelicheva, 2011). Such framing provides fertile ground for the imposition of negative identities on groups that practice what is often labeled “non-traditional Islam” or that belong to new religious movements.

### 1.3 Post-secular theories and religion in the public sphere

In Kazakhstan, these boundary-making dynamics also intersect with state-led processes through which national belonging and collective identity are rhetorically constituted and linked to political legitimation (Cummings, 2006; Rees and Burkhanov, 2018), as well as with scholarship on state–confessional relations and the political management of religious trends in Kazakhstan’s secular institutional framework (Aymukhambetov and Rysbekova, 2016; Burova et al., 2020).

Post-secular theory, developed by Habermas (2006) and other scholars (Taylor, 2007), offers an alternative account of religion’s place in contemporary societies. From this perspective, religion does not disappear from the public sphere; rather, it is reconfigured, assuming new forms of visibility, participation, and influence.

Applied to Kazakhstan, a post-secular lens makes it possible to examine how the state and society construct a hierarchy of religious identities by privileging “traditional” religions (Hanafi Islam and Orthodoxy) while framing other denominations as potentially dangerous or as alien to national culture.

### 1.4 Religious stigma as an ideological instrument: theory and mechanisms

#### 1.4.1 Ideology and the construction of “others”

Any ideology requires the demarcation of the community to which it is addressed and the identification of “others” positioned beyond its boundaries (Althusser, 1971). In the nation-building processes of post-Soviet states, religious identities have become a key marker for determining who belongs to the national community and who may be positioned outside state-endorsed understandings of legitimate belonging (Dave, 2007; Laruelle, 2018; Yemelianova, 2014).

According to Louis Althusser’s critical theory of ideology, ideology operates through “interpellation” – a process through which individuals are hailed into subject positions (Althusser, 1971). In the religious domain, state ideology interpellates citizens as bearers of specific religious identities: “traditional Muslims,” “Orthodox Christians,” or, conversely, “suspect non-believers,” “radicals,” or “sectarians.”

Antonio Gramsci conceptualized ideology as an instrument for establishing hegemony – dominance achieved not primarily through direct coercion, but through the production of “common sense” and widely shared norms (Gramsci, 1971). In the Kazakhstani context, ideological hegemony becomes visible in the naturalization of a religious hierarchy, whereby the privileged status of “traditional” confessions and the stigmatization of “non-traditional” ones are perceived not as the outcome of political choice, but as a self-evident and “natural” order of things.

### 1.5 The ideological functions of religious stigmatization

The stigmatization of specific religious groups performs several key ideological functions in post-Soviet Kazakhstan:

1. Demarcating the boundaries of the national community

In the context of post-Soviet transformation, when prior ideological foundations have been dismantled, religion becomes an important marker of national identity. Yet not all forms of religiosity are recognized as compatible with Kazakhstani identity. “Traditional” religions are incorporated into national ideology as elements of cultural heritage, whereas “non-traditional” denominations and currents are constructed as alien and as threatening to national identity (Omelicheva, 2011; Yemelianova, 2014).

This logic reflects a broader ideological project in which the Kazakhstani nation is defined not in narrowly ethnic terms, but through a cultural–civilizational framework. Adherence to a “proper” form of religiosity becomes a marker of belonging to the national community, while “improper” religiosity serves as a basis for exclusion.

1. Legitimizing state control

The construction of religious groups as security threats legitimizes the expansion of state control not only over these groups, but over the religious sphere (Omelicheva, 2011). The ideology of securitization transforms issues of religious freedom into issues of national security, rendering restriction and oversight not merely permissible, but necessary.

This dynamic resonates with what Michel Foucault termed “governmentality” – a mode of governing populations through the production of kinds of subjects and forms of knowledge about them (Foucault, 1991). Categorizing religious groups as “traditional” versus “non-traditional,” or “moderate” versus “radical,” produces a classificatory knowledge regime that enables – and legitimizes – differentiated governance of diverse religious communities.

1. Mobilizing consensus around national ideology

Constructing the image of a religious “other” – whether the “radical Islamist” or the “destructive sect” – may facilitate public consolidation around state-defined understandings of security and legitimate religious belonging (Laruelle, 2013; Omelicheva, 2011).

The high level of public support for state religious policy (69.7%; Center for Social and Political Studies “Strategy”, 2023) is consistent with this ideological strategy. The sample size, sampling method, and margin of error for this survey are not published alongside the figure and could not be independently verified for this study; the finding is therefore reported here as suggestive rather than conclusive. If it holds, it would suggest that most of the population views the regulation of “dangerous” religious groups as necessary, which would reinforce the state’s claim to legitimacy as protector of national interests – an interpretation developed further in the Discussion (§4.1.3).

1. Managing ideological contradictions

Official narratives of Kazakhstan as a multiethnic and multi-confessional state have emphasized social unity and managed pluralism, frequently positioning “traditional” confessions as stabilizing cultural resources (Nazarbayev, 2014). At the same time, critical debates on ideology caution against the political effects of overly rigid classificatory schemes and totalizing distinctions (Arendt, 2011).

Post-Soviet Kazakhstan navigates tensions between modernization and the reinterpretation of national cultural traditions, as well as between secular state principles and the renewed public role of religion (Koyanbaeva and Zaurbekova, 2021; Mukan et al., 2023).

Constructing a hierarchy of religions allows these tensions to be partially managed. “Traditional” religions – particularly Hanafi Islam – are represented as compatible with modernization: moderate, rational, and socially integrative. “Non-traditional” currents, by contrast, are framed as archaic, irrational, or excessively fanatical. This enables religion to be retained as an element of national identity while rejecting those forms of religiosity that are portrayed as incompatible with the modernization project.

### 1.6 The ideology of “traditionalism” and the construction of religious normativity

A central element of Kazakhstan’s state ideology in the religious sphere is the concept of “traditionalness” (tradition-based legitimacy). This category performs crucial ideological work by naturalizing a particular form of religiosity as the only authentic and socially acceptable one.

Yet, as critical scholarship has demonstrated, “tradition” itself is an ideological construction (Hobsbawm and Ranger, 1983). What is presented today as “traditional Islam” is, in important respects, the product of a specific historical trajectory shaped by Soviet nationalities policy, which established institutional infrastructures (such as Muslim Spiritual Boards) and fostered modalities of religious practice (Montgomery, 2016; Yemelianova, 2014).

Moreover, the very category of “traditionalness” is applied selectively. Practices that historically formed part of the region’s religious life (Sufi orders, vernacular or popular forms of Islam) may today be labeled “non-traditional” when they do not conform to a state-sanctioned model of religiosity (Privratsky, 2001; Yemelianova, 2014). The ideology of traditionalism thus serves less to preserve historical continuity than to construct a politically usable version of the past – one that legitimizes contemporary configurations of power and the hierarchy of religious identities.

### 1.7 Transnational ideological conflicts

Globalization in the religious sphere means that believers in Kazakhstan increasingly interact with religious ideas, authorities, and communities extending beyond nationally regulated institutional structures. In the Central Asian context, these transnational connections contribute to the diversification of religious practices, identities, and sources of authority and may complicate state efforts to define a nationally legitimate model of religiosity (Laruelle, 2018; Balci, 2018; Peyrouse, 2010).

From the standpoint of national ideology, such transnational religious identities are perceived as threatening precisely because they propose alternative sources of authority and belonging that compete with the nation-state. A Muslim whose primary self-understanding is rooted in membership in the global umma, or a Christian whose primary loyalty is directed toward a transnational religious organization, becomes potentially problematic for an ideological framework premised on the primacy of national identity.

In this sense, the discourse of “foreign influence” in the religious sphere reflects not only concerns about the geopolitical interests of external actors, but a deeper ideological conflict between national and transnational forms of identity and loyalty.

### 1.8 The ideological struggle for youth

For contextual triangulation, the analysis also draws on recent Kazakhstan-based sociological research on religious identity, which examines how religious affiliation intersects with social positioning and socioeconomic differentiation (Abdiraiymova and Saimassayeva, 2023).

The particular attention that state ideology pays to youth religiosity reflects an awareness that the future of ideological hegemony depends on shaping the consciousness of the next generation. Young people are framed simultaneously as especially vulnerable to “ideological processing” by “destructive” religious movements and as the most strategically important audience for the transmission of state ideology.

Secondary survey data report that 41.5% of young respondents regularly perform the five daily prayers and that 46.2% adhere to dress practices described in the source as “sharia-compliant.” Because the available source does not report the sample size, sampling procedure, or margin of error, these figures should be treated as descriptive rather than as evidence of an increase over time (Center for Social and Political Studies “Strategy”, 2023). At the same time, some young people form their religious identities through digital and transnational networks, bypassing traditional institutional structures that are more easily monitored and regulated by the state.

This produces an ideological dilemma: how can religiosity be encouraged as an element of national identity while simultaneously controlling the forms that religiosity takes? State ideology attempts to manage this contradiction by promoting “proper” religiosity and stigmatizing “improper” variants. Yet the effectiveness of this strategy is constrained under conditions of digitalization and the globalization of the religious sphere.

## 2 Methodology

The study of imposed religious identities in Kazakhstan is grounded in an interdisciplinary approach that integrates sociological, political-science, and religious-studies methods. The methodological framework comprises several key components.

Study design and case selection. This article adopts an interdisciplinary case-study design focused on Kazakhstan’s post-Soviet religious landscape. It combines descriptive analysis of secondary quantitative data with qualitative analysis of policy documents, legal materials, and media discourse. The case is selected because Kazakhstan represents a high-pluralism, formally secular context where religion has become a salient marker in projects of national identity and governance.

Qualitative corpus and source-level review. The qualitative component comprised the Constitution of the Republic of Kazakhstan, the 2011 Law “On Religious Activities and Religious Associations,” related policy documents, and six publicly accessible media and official materials published between 2016 and 2023 and listed in Table 1. The six materials were identified through a targeted search conducted in August 2026 on gov.kz, the official website of the National Security Committee, and Kazinform. Russian-language search combinations corresponding to “traditional religions,” “non-traditional religious movements,” “religious extremism,” “foreign influence,” “pseudoreligious organizations,” “youth,” and “social networks” were used. Materials were included if they were publicly accessible, had an identifiable publisher and publication date, and contained a clear example of one of the analytical frames examined in the study. The sample was purposive and illustrative and was not used to estimate the frequency or prevalence of these frames.

**Table 1:** Illustrative examples of qualitative frames in publicly accessible media and official materials.

Qualitative frame	Group or issue represented	Illustrative wording or context	Source and date
Cultural heritage and social stability	Hanafi Islam, Orthodox Christianity, and interconfessional relations	Hanafi Islam and Orthodox Christianity are positioned as religions historically rooted in Kazakhstan, while interconfessional diversity is connected with social harmony, stability, peace, and national security.	*Main Priorities of Kazakhstan’s State Policy in the Religious Sphere*, 10 March 2022 (Department for Religious Affairs of the Karaganda Region, 2022).
Traditional/non-traditional boundary	Islam and Orthodox Christianity contrasted with newer religious movements	Islam and Orthodox Christianity are described as historically connected with national culture, spiritual life, customs, and traditions, whereas “non-traditional” religions are presented as comparatively recent and associated with missionary and proselytizing activity.	*Traditional and Non-Traditional Religions*, 13 March 2023 (Akimat of Denisov District, 2023).
Foreign influence	Foreign religious centers and transnational religious actors	The location of religious centers outside Kazakhstan and the activity of transnational religious organizations are presented as channels through which external religious influence may enter the domestic religious sphere.	*Religion and the State*, 17 May 2016 (Kazinform, 2016).
Sectarian manipulation and social destabilization	Organizations characterized as “pseudoreligious” or “destructive”	The material uses the language of manipulation, destruction of established values, social destabilization, and geopolitical, financial, or ideological objectives when discussing pseudoreligious organizations.	*Pseudoreligious Organizations: What Makes Them Dangerous and How They Operate on Social Networks*, 27 April 2022 (Department of Religious Affairs of Almaty, 2022).
Youth vulnerability	Young people and socially vulnerable groups	Young people aged approximately 17–35 and socially vulnerable individuals are represented as particularly susceptible to radical or destructive religious influence because of limited religious knowledge and personal vulnerability.	*Young People under 35 Are Most Frequently Exposed to Radical Influence*, 8 September 2020 (Kazinform, 2020).
Digital radicalization and preventive intervention	Young users of social networks	Radical ideology disseminated through social networks is framed as a particular risk for young people, accompanied by recommendations concerning parental attention, avoidance of extremist online resources, and preventive intervention.	*On the Detention of a Radical*, 27 December 2023 (National Security Committee of the Republic of Kazakhstan, 2023).

The table presents purposively selected source-level illustrations identified through a targeted source-level review of publicly accessible media and official materials. The “illustrative wording or context” column paraphrases the representational framing found in the respective source and does not reproduce direct quotations. Inclusion in the table does not imply endorsement of the descriptions used in the source and does not establish the accuracy, frequency, or prevalence of the identified frames across Kazakhstani media or official discourse as a whole. Full bibliographic details are provided in the reference list. Sources: Akimat of Denisov District (2023); Department of Religious Affairs of Almaty (2022); Department for Religious Affairs of the Karaganda Region (2022); Kazinform (2016, 2020); National Security Committee of the Republic of Kazakhstan (2023). Two materials hosted on gov.kz reproduce attributed expert or media content and are analyzed as examples of publicly hosted discourse rather than as binding statements of official government policy.

Analytic strategy. Media and policy texts were examined through qualitative thematic analysis and discourse analysis. The analysis identified recurring labels, frames, and associations used to describe religious groups, including “traditional,” “non-traditional,” “sect,” “radical,” and “foreign influence,” and considered how these categories delineate the boundaries of the national community and legitimize differentiated regulation. The analytic categories were informed by the theoretical framework and refined through close reading of the materials. Because no fully documented article-level coding protocol was retained, the analysis does not report frame frequencies or inter-coder reliability. Instead, Table 1 provides a set of source-level examples to make the qualitative interpretation transparent and independently traceable.

Validity and limitations. The study triangulates across statistical trends, legal–policy language, and media framing to strengthen interpretive validity. At the same time, the article relies on publicly available materials and secondary survey reporting; it does not include primary interviews with members of stigmatized groups, which limits access to lived experience and micro-level mechanisms of coping. These limitations are addressed in the Discussion as directions for future research.

### 2.1 Research design

The study uses an interdisciplinary case-study design combining descriptive secondary statistics with qualitative document and media analysis.

### 2.2 Data sources

Quantitative data:

- Kazakhstan’s two most recent national population censuses (2009 and 2021), including information on the population’s religious affiliation;
- Statistics on registered religious associations published by the Ministry of Culture and Information of the Republic of Kazakhstan, as reported by Kazinform (2024);
- Findings from nationwide sociological surveys conducted by the Center for Social and Political Studies “Strategy” (2023);
- Survey data on religious practice and identity across selected cities of Kazakhstan.

Qualitative data

- Regulatory and legal acts governing the religious sphere (the Constitution of the Republic of Kazakhstan; the Law “On Religious Activity and Religious Associations”; strategic documents on state religious policy);
- Six publicly accessible media and official materials published between 2016 and 2023 and reported at source level in Table 1.

### 2.3 Methods of analysis

Statistical analysis was used to identify quantitative trends in changes to the confessional composition of the population, the dynamics of religious self-identification, and regional differences in levels of religiosity. Comparative analysis of census data made it possible to trace transformations in the confessional landscape during the post-Soviet period.

Qualitative thematic analysis of media materials and official documents was used to identify recurring patterns in the representation of different religious groups, including the contexts in which they appeared and the descriptors and associative frames applied to them. Because the media corpus was not coded using a fully documented frequency-based protocol, the analysis focused on recurrent qualitative patterns rather than statistically estimated prevalence.

Discourse analysis of regulatory/legal acts and programmatic documents was applied to reconstruct the state’s official stance toward different religious groups, identify an implicit hierarchy of confessions within state policy, and examine mechanisms through which differential treatment of religious organizations is legitimized.

Finally, regional survey data were used descriptively to compare variation in religious self-identification across selected cities, without making causal or correlational claims.

## 3 Results

### 3.1 Kazakhstan’s religious landscape and the ideological hierarchy of identities

#### 3.1.1 Statistical profile of religious diversity

An examination of official population statistics indicates a marked transformation of Kazakhstan’s religious landscape in the post-Soviet period. According to Kazakhstan’s two most recent national censuses (2009 and 2021; no national census has been conducted since 2021), the share of the population identifying as Muslim was 70.20% in 2009 and 69.31% in 2021, the Christian share fell from 26.17% to 17.19%, the share identifying as atheist declined from 2.81% to 2.25%, and the share declining to state a religious affiliation rose sharply from 0.51% to 11.01% (Agency of the Republic of Kazakhstan on Statistics, 2010; Bureau of National Statistics of the Republic of Kazakhstan, 2023). A frequently cited figure of 92.8% self-identifying as religious originates from a separate 2019 sociological survey rather than from census enumeration and is therefore not directly comparable to the census-based percentages above; the two should not be conflated when reporting a single summary statistic (Kazakhstan Institute for Strategic Studies, 2019, as cited in U.S. Department of State, 2023). Taken together, the census and survey sources do not support a single estimate of the growth of religiosity because they measure different phenomena and use different methods. The census data show relative stability in Muslim self-identification, a decline in Christian identification, a modest decline in atheism, and a marked increase in non-response between 2009 and 2021. The sharp increase in non-response is analytically important but substantively ambiguous; the available census data do not permit direct conclusions about respondents’ motivations. Table 2 reports the underlying census figures in full.

**Table 2:** Religious self-identification of the population of Kazakhstan, 2009 and 2021 censuses (%).

Category	2009 (%)	2021 (%)
Muslim	70.20	69.31
Christian	26.17	17.19
Atheist / no religion	2.81	2.25
Other religious affiliations	0.31	0.24
Declined to state religious affiliation	0.51	11.01

Agency of the Republic of Kazakhstan on Statistics (2010); Bureau of National Statistics of the Republic of Kazakhstan (2023). The 2009 figures are drawn from the results of the 2009 National Population Census, while the 2021 figures are drawn from the thematic collection of the 2021 National Population Census. “Other religious affiliations” combines the census categories of Judaism, Buddhism, and other religious affiliations. “Declined to state religious affiliation” is reported as a separate category. Percentages may not sum exactly to 100% because of rounding.

According to the 2021 census, Muslims account for approximately 69% of the population, Christians for approximately 17%, atheists for approximately 2%, while 11% reported difficulty answering or declined to specify their religious affiliation (Bureau of National Statistics of the Republic of Kazakhstan, 2023; see Table 2). From an ideological perspective, this latter category warrants particular attention. The census data do not identify respondents’ reasons for non-response. The category may reflect several different factors, including uncertainty, privacy concerns, multiple religious identification, sensitivity of the question, or, in some cases, an effort to avoid potentially stigmatizing disclosure.

An additional, complementary indicator of Kazakhstan’s religious landscape is the number of religious associations formally registered with the state, which captures institutional presence rather than self-reported belief and is therefore not directly comparable to the census figures in Table 2, but offers a concrete, checkable measure of confessional diversity referenced throughout this article (cf. §1.4, §3.1.2) (see Table 3).

**Table 3:** Religious associations registered in Kazakhstan, by confession (2023).

Confession / group	Registered associations (2023)
Islamic	2,835
Orthodox Christian	347
Protestant, total (of which:)	586
Pentecostal	222
Baptist	186
Presbyterian	104
Seventh-day Adventist	40
Lutheran	14
Methodist	11
Mennonite	9
Roman Catholic	93
Jehovah’s Witnesses	61
New Apostolic Church	24
International Society for Krishna Consciousness	13
Jewish	7
Baha’i	6
Buddhist	2
Church of Jesus Christ of Latter-day Saints	2
Unification Church	1
Total (18 confessions)	3,977

Ministry of Culture and Information of the Republic of Kazakhstan, as reported by Kazinform (2024).

Regional survey data further indicate that self-assessed religiosity varies considerably by city, which qualifies national-level averages reported elsewhere in this article (§1.8, §4.1.2) and is presented here as descriptive detail rather than as a basis for causal claims about regional determinants.

The regional variation reported in Table 4 does not in itself demonstrate religious stigmatization. It is included to show that national-level averages conceal substantial differences in religious self-positioning across urban contexts. Because the available secondary source does not report the sample size, sampling procedure, or margin of error, no causal or statistically generalizable inference is drawn from these comparisons.

**Table 4:** Self-assessed religiosity by city, survey responses (%).

Category	Almaty	Astana	Karaganda	Kostanai	Taraz	Ust-Kamenogorsk	Aktobe
Not religious, not interested	12	14	8	13	6	9	7
Interested in religion, but do not consider self-religious	16	27	47	32	28	27	30
Consider self-religious, do not practice	37	38	36	38	31	43	30
Consider self-religious, practice regularly	28	21	6	14	35	18	30
Difficulty answering	7	0	3	3	0	3	3

Center for Social and Political Studies “Strategy” (2023). Rows may not sum to 100% due to rounding. Sample size, sampling method, and margin of error for this survey are not published in the secondary sources available to the authors (see Limitations, §4.3).

#### 3.1.2 The hierarchy of religious identities in state discourse

An analysis of the Constitution of the Republic of Kazakhstan, the 2011 Law “On Religious Activities and Religious Associations,” and related policy documents suggests an implicit hierarchy of religious identities within state discourse. While the Constitution affirms the secular character of the state and guarantees freedom of religion, the preamble to the 2011 Law explicitly recognizes the historical role of Hanafi Islam and Orthodox Christianity in the development of Kazakhstan’s cultural and spiritual life, while referring more generally to respect for other religions (Republic of Kazakhstan, 1995, 2011). On this basis, the following three-tier structure is used as an analytical interpretation of the reviewed documents rather than as a formal legal classification.

Within official discourse, three categories of religions can be identified:

1. “Traditional” religions – Hanafi Islam and Orthodox Christianity – which are integrated into national ideology as integral elements of cultural heritage and enjoy a privileged status. These confessions are presented as historically rooted, compatible with Kazakhstani identity, and conducive to social stability.
2. “Historically present” confessions – Catholicism, Protestantism (in its established forms), Buddhism, and Judaism – which are recognized as part of the country’s religious landscape but occupy an intermediate position within the ideological hierarchy.
3. “New” or “non-traditional” movements – including newer Protestant denominations, Jehovah’s Witnesses, the International Society for Krishna Consciousness, and other non-traditional currents within Islam. These groups are ideologically constructed as potentially dangerous, alien to national culture, and suspect regarding loyalty to the state.

The three-tier structure presented above constitutes an analytical classification developed by the authors on the basis of the reviewed legal and policy documents; it is not explicitly codified as such in Kazakhstan’s legislation.

#### 3.1.3 Constructing “suspicious” religious identities

The six purposively selected media and official materials illustrate several qualitative associations between religious groups and themes of cultural heritage, security, extremism, foreign influence, manipulation, and youth vulnerability. Among the qualitative patterns identified in the reviewed materials were the positive cultural-heritage framing of “traditional” religions, particularly Hanafi Islam and Orthodox Christianity; the security-oriented framing of some Islamic currents categorized as “non-traditional”; and references to new religious movements through the language of “sects,” manipulation, foreign influence, and youth vulnerability. Young people whose religious identities are shaped through digital platforms and transnational religious networks were also represented in some materials as particularly susceptible to radicalization and therefore in need of protection or preventive intervention. These findings are reported as recurring qualitative patterns within the purposively selected corpus rather than as estimates of their statistical frequency or prevalence across Kazakhstani media as a whole.

To provide source-level transparency, Table 1 presents six illustrative examples identified through the targeted source-level review of publicly accessible media and official materials described in the Methodology (Department for Religious Affairs of the Karaganda Region, 2022; Akimat of Denisov District, 2023; Kazinform, 2016, 2020; Department of Religious Affairs of Almaty, 2022; National Security Committee of the Republic of Kazakhstan, 2023). These materials demonstrate how the qualitative frames discussed above appeared in specific texts. The examples document the language and context of representation; they are not treated as evidence that the represented groups necessarily possess the characteristics attributed to them.

The examples in Table 1 provide traceable source-level evidence of how the identified qualitative frames appeared in specific texts. They show that positive cultural-heritage representations of “traditional” religions coexist with security-oriented, foreign-influence, manipulation, and youth-vulnerability frames applied to other religious actors and practices. Because this purposive sample was selected for analytical relevance, no claim is made regarding the statistical prevalence of these frames across Kazakhstani media or official discourse as a whole.

Institutional efforts to promote interconfessional dialogue and social cohesion – including initiatives documented by the Assembly of the People of Kazakhstan – form part of the policy environment within which religious diversity is publicly managed and represented (Assembly of the People of Kazakhstan, 2015).

Taken together, these patterns are consistent with the theoretical expectation that confessional affiliation can become a marker of suspicion in public discourse. Their broader ideological implications are examined in the Discussion.

## 4 Discussion

### 4.1 Potential implications of imposed identities for stigmatized groups

#### 4.1.1 Potential psychological and social implications

Although this study did not collect primary data on the lived experiences of members of religious groups that are subject to stigmatizing classifications, the theoretical framework and secondary evidence allow us to outline the principal pathways through which externally assigned identities may shape everyday interactions.

In line with Goffman’s account of stigma (Goffman, 1963), individuals who are aware that their group is negatively evaluated in the public sphere may experience heightened uncertainty in interactions with members of the majority. They may engage in various forms of information management regarding religious affiliation – deciding whether to disclose it, in which contexts, and to whom, and anticipating possible social consequences.

In the context of religious minorities in Kazakhstan, these dynamics may take several forms:

1. Identity management and limited public visibility – reducing outward markers of affiliation (e.g., avoiding visible religious symbols, limiting attendance at public religious events, or keeping religious materials private). According to the 2021 national census, 11.01% of respondents did not state a religious affiliation. The census data do not establish the reasons for non-response; possible explanations may include uncertainty, privacy concerns, multiple identification, the sensitivity of the question, or identity-management strategies in socially sensitive contexts.
2. Psychological strain – concerns about negative judgment, the anticipation of discriminatory treatment, and the experience of social pressure. Research on stigma in other settings suggests that sustained exposure to stigmatizing environments may be associated with higher levels of stress and anxiety.
3. Adaptive self-restriction – selective decisions regarding educational and professional trajectories, reduced public participation, or narrower social networks, motivated by expectations of misunderstanding or unequal treatment.

#### 4.1.2 Courtesy stigma and effects on families and social networks

The notion of courtesy stigma highlights that social discrediting can extend to those who are closely associated with members of a stigmatized group (Goffman, 1963). In religious contexts, this implies that family members may also encounter negative labeling or social distancing.

This issue may be particularly salient in Kazakhstan, where family and community ties play a significant role in social life. In such settings, the religious affiliation of one family member may:

- affect marriage-related considerations within the broader family network;
- influence social and professional relationships of relatives;
- shape perceptions within local communities;
- generate intra-family tensions, especially where religious orientations differ across generations.

Secondary survey data report similar levels of regular performance of the five daily prayers among the overall sample (41.8%) and youth (41.5%) (Center for Social and Political Studies “Strategy”, 2023). However, because the available source does not provide the sample size, sampling procedure, or margin of error, these figures should be treated as descriptive rather than as precise population estimates. Existing scholarship further suggests that younger cohorts may be more likely to rely on digital sources and transnational networks when forming religious views, which can complicate communication within families oriented toward locally established or institutionally mediated religious norms.

#### 4.1.3 Media and state policy in the production of religious stigma

As reported in Results (§3.1.3), the six purposively selected media and official materials illustrate differences in the representation of religious groups. These patterns have two interrelated implications. First, they may reinforce public support for regulatory approaches to the religious sphere by presenting such measures as preventive or protective. Second, they may contribute to social distance toward particular groups and create conditions in which exclusionary practices become normalized.

Analysis of legislation and policy documents suggests that Kazakhstan’s approach may be described as managed pluralism: a combination of formal commitments to freedom of religion with differentiated institutional expectations and regulatory attention across confessions.

The reported figure of 69.7% support for state regulation of the religious sphere (Center for Social and Political Studies “Strategy”, 2023) is treated here as suggestive secondary evidence because the available source does not report the sample size, sampling design, or margin of error. The figure may be consistent with a broader preference for stability and regulatory oversight, but it cannot independently establish the normalization of a confessional hierarchy.

### 4.2 Transnational religious networks: resource or risk?

Transnational religious networks can play an ambivalent role in the formation of religious identities in Kazakhstan. They may provide access to alternative religious knowledge, communities, and sources of authority, while also attracting intensified regulatory attention when they are represented as channels of external ideological influence (Laruelle, 2018; Balci, 2018; Peyrouse, 2010). On the one hand, such networks can provide religious communities with organizational, informational, and symbolic resources that support community development and help mitigate stigmatizing pressures. On the other hand, ties to foreign-based religious organizations are often invoked in public and policy discourse as a rationale for intensified oversight and, in some cases, for the reinforcement of stigmatizing classifications.

The digitalization of religion has further reshaped processes of religious identity formation, particularly among younger cohorts. Online platforms and social media expand access to global religious authorities, enable believers to circumvent established institutional channels, and facilitate identity construction through individualized choice and self-directed learning.

At the same time, digital religiosity can generate additional forms of vulnerability. State regulation of online space, monitoring of religious content on social media, and the criminalization of the dissemination of materials designated as “extremist” (a category that may be defined expansively in practice) can create added risks for individuals who develop and express religious identities through digital platforms.

Survey findings indicate that only 7.8% of young respondents reported coming to faith “with the help of preachers,” whereas 45.8% stated they had been “introduced by parents from childhood,” and 23.7% reported doing so “through a sudden insight” (Center for Social and Political Studies “Strategy”, 2023). Because the available source does not provide full methodological documentation, these figures should be interpreted descriptively. They suggest that family socialization and individualized experience may be more salient than direct preacher involvement, but they do not by themselves demonstrate either a declining role of institutional mediation or an increasing reliance on digital environments.

The findings are consistent with scholarship on stigma as a social mechanism that transforms group membership into a discrediting attribute and produces predictable strategies of concealment and impression management (Goffman, 1963; Kellezi et al., 2019). In the Kazakhstani context, however, stigma operates not only through interpersonal labeling but also through institutionalized classifications embedded in policy language and media frames. This supports the argument that imposed religious identities should be understood as an ideological instrument – one that organizes the moral boundaries of the national community and renders differential governance intelligible.

Contribution to debates on ideology and governance. Building on the theoretical framework set out in §1.4 (Althusser, 1971; Gramsci, 1971; Foucault, 1991) rather than restating it, the present results show these categories at work: security discourse functions as the vehicle through which the “traditional/non-traditional” boundary is stabilized, and this classification legitimizes differentiated regulation of specific groups.

Securitization as boundary-making. The study illustrates how securitization reconfigures religion into a security object, enabling heightened regulatory attention toward particular groups and practices (Omelicheva, 2011). Notably, the category of “non-traditional Islam” functions as a shorthand that can compress diverse theological and social realities into a single risk-signifier, thereby amplifying suspicion and facilitating broad, preventive governance (Naumkin, 2005). This framework offers one possible interpretation of census non-response, but the available data do not establish respondents’ motivations. Non-response may reflect privacy concerns, uncertainty, multiple identification, sensitivity of the question, or, in some cases, efforts to avoid stigmatizing disclosure.

Transnational religion and the politics of loyalty. The section on transnational networks shows that cross-border religious ties can operate both as resources for minority communities and as triggers for “foreign influence” narratives. This suggests that the issue for national ideology may concern not religiosity per se, but the emergence of religious authorities and forms of belonging that extend beyond nationally regulated institutional structures (Laruelle, 2018; Balci, 2018; Peyrouse, 2010). Under conditions of digitalization, these dynamics intensify: individualized religious learning via online spaces can bypass domestically sanctioned institutions, complicating attempts to channel religiosity into officially preferred forms.

Kazakhstan-specific implications. Official narratives of Kazakhstan as a multiethnic, multi-confessional state emphasize unity and social cohesion, often positioning “traditional” confessions as stabilizing cultural resources (Nazarbayev, 2014). The present findings suggest that this model can coexist with a durable hierarchy of confessions in which certain groups are socially and administratively “problematized.” As a result, pluralism may take a managed form that protects interfaith harmony at the level of official symbolism, while reproducing differentiated treatment at the level of public categorization and regulatory practice (Burova et al., 2020; Omelicheva, 2011).

### 4.3 Limitations and future research

Three limitations are central. First, the study relies on public texts and secondary reporting rather than direct interviews with members of stigmatized communities, which constrains the analysis of lived experience, coping, and the micro-dynamics of “courtesy stigma.” Second, the media and official-text component is based on six purposively selected source-level illustrations. Although this approach improves transparency and traceability, it does not establish the frequency, distribution, or representativeness of the identified frames across Kazakhstan’s wider media environment. No inter-coder reliability statistic or frame-frequency analysis is reported because the analysis is qualitative and illustrative rather than frequency-based. Third, the secondary survey figures drawn from the Center for Social and Political Studies “Strategy” were available through the published institutional report, but the underlying microdata and full methodological documentation were not available to the authors. Consequently, the precision and generalizability of these survey-based percentages cannot be independently assessed, and the corresponding findings should be interpreted as descriptive and indicative rather than as precisely estimated population parameters. Further research could combine interviews and ethnographic observation with computational text analysis of media and social-media content, allowing stronger causal claims about how stigmatizing frames circulate and how they shape behavior, trust, and civic participation. Comparative work across Central Asian states would also help distinguish which mechanisms are shared regionally and which are specific to Kazakhstan’s institutional model. More broadly, historical scholarship reminds us that the politicization of religious boundaries has varied forms across time and place, underscoring the value of careful comparison without direct equivalence.

## 5 Conclusion

This study suggests that externally imposed religious identities in Kazakhstan operate within a broader system of social categorization and differentiated representation. Despite constitutional guarantees of freedom of religion and official support for religious pluralism, the analyzed policy, statistical, and media materials indicate the persistence of a confessional hierarchy in which certain groups are more readily framed through security, foreign-influence, or social-risk narratives.

More broadly, the Kazakhstani case reflects a wider post-Soviet configuration of state–religion–society relations. While religious pluralism is formally affirmed, hierarchical distinctions and exclusionary mechanisms may continue to be reproduced in practice, generating patterns that resemble what has been described as a “social curse” for groups.

Addressing these dynamics would require not only legal and policy adjustments, but also more far-reaching shifts in public attitudes, media practices, and educational approaches. Substantive religious pluralism entails more than the formal recognition of freedom of conscience; it also involves creating social conditions under which members of diverse religious traditions can participate fully in public life without anticipating stigmatization or unequal treatment.

Kazakhstan’s experience suggests that even in societies with longstanding traditions of religious and ethnic diversity, stigmatizing processes can create durable barriers to social integration and the effective realization of rights. Recognizing these dynamics and engaging in sustained efforts to address them constitutes an important condition for building a more inclusive and pluralistic society.

## Data Availability

The study draws on publicly available census, administrative, legal, policy, media, and official sources. The six source-level materials analyzed in Table 1 are publicly accessible and fully cited in the reference list. Secondary survey figures were taken from the cited institutional report; the underlying microdata and full methodological documentation were not available to the authors. Further inquiries can be directed to the corresponding author.
